# Solvent-free synthesis of NiCo_2_S_4_ having the metallic nature

**DOI:** 10.3389/fchem.2022.1027024

**Published:** 2022-10-21

**Authors:** Sardar Ahmed, Mushtaq Ahmad, Muhammad Hasnain Yousaf, Sumain Haider, Zahid Imran, S. S. Batool, Ishaq Ahmad, Muhammad Imran Shahzad, Muhammad Azeem

**Affiliations:** ^1^ Catalysis and Sensing Materials Group, Department of Physics, COMSATS University Islamabad, Islamabad, Pakistan; ^2^ Department of Physics, The University of Hong Kong, Pokfulam, Hong Kong SAR, China; ^3^ Department of Chemistry, University of Sialkot, Sialkot, Pakistan; ^4^ Nanosciences and Technology Department (NS&TD), National Centre for Physics (NCP), Islamabad, Pakistan; ^5^ Department of Applied Physics and Astronomy, University of Sharjah, Sharjah, United Arab Emirates

**Keywords:** solvent-free synthesis, solid state reaction, metallic behavior, x-rays diffraction, transmission election microscopy, current—voltage (I-V) characteristic

## Abstract

Nickel-cobalt sulfide (NiCo_2_S_4_) is a prominent member of bimetallic transition metal sulfides. It is being widely used for a variety of applications such as electrode material, photocatalysis, and energy storage devices (like pseudo capacitors, supercapacitors, solar cells, and fuel cells) due to its better electronic conductivity, manageable morphology, and high capacitance. This work presents the one-step solventless synthesis of NiCo_2_S_4_ sheet-like nanostructures and then explores their metallic nature. Scanning electron microscopy (SEM) and transmission electron microscopic (TEM) analysis show the sheet-like grown morphology. Few nanorods are also seen. Except for a recent study (Xia et al. 2015) that shows metallic behavior, most of the reports show that NiCo_2_S_4_ is a semiconductor with claimed bandgap between 1.21 and 2.4 eV. In this study, we observe from UV-Vis and diffuse reflectance spectroscopy (DRS) that NiCo_2_S_4_ has a specific band gap value between 2.02 and 2.17 eV. However, IV characteristics in the temperature range of 300–400 K show that NiCo_2_S_4_ is a metal with a positive temperature coefficient of resistance consistent with a recent report. Furthermore, we see the ohmic conduction mechanism. The Arrhenius plot is drawn, and the activation energy is calculated to be 3.45 meV. The metallic nature is attributed to the coupling of two metal species (nickel and cobalt), which accounts for its superior conductivity and performance in a variety of essential applications.

## Introduction

NiCo_2_S_4_ has sparked a lot of attention in recent years, and it is being studied as a potential material for a variety of applications because of its fascinating characteristics. It is one of the important members of the bimetallic transition metal sulfides. Transition metal compounds have been known for unique properties, like inexpensive, pt.-like catalytic performance, large conductive, etc. (Guan et al., 2017; Chia et al., 2015; Tong et al., 2018). Among the bimetallic compounds, the NiCo_2_S_4_ has a smaller optical energy bandgap and much better electronic conductivity than nickel cobalt oxides and hydroxides counterparts (Chen et al. 2013; Zhang et al., 2014). The NiCo_2_S_4_ recently achieved remarkable performance in energy storage devices like electrode material in supercapacitors (Zhu et al. 2015; Gao and Huang, 2017; Zheng et al., 2018), catalysis (Zhang et al., 2014; Wu et al., 2018; Wang et al., 2019), and dye-sensitized solar cell (Lin and Chou, 2013; Yang et al., 2014). Most studies on NiCo_2_S_4_, like other chalcogenides, establish that it is semiconducting, with a claimed bandgap between 1.2 and 2.4 eV (Chen et al. 2013; Du et al., 2014; Sarawutanukul et al., 2020). However, a very recent study (Xia et al., 2015) has proven that this material is behaving like a metal, based on optical and electrical results. They have reported that at room temperature the resistivity of NiCo_2_S_4_ nanostructures is around 10^3^ μΩ cm, which then decreases with the decrease in temperature. It denotes a positive temperature coefficient of resistance, indicating the conducting nature of the NiCo_2_S_4_ nanostructures. Moreover, it is important to mention the effects of cations distributions in the bimetallic sulfides because the variation in cations influences the electroactive nature of the material for energy generation. Although, the substitution of cobalt with nickel and *vice versa* does not change the crystal structure of the compound (Chen et al., 2013), but cobalt-rich presence as compared to nickel adds more holes (*p-*type) i.e., makes less conductive material and when there is more presence of nickel, the material gets more electrons (*n-*type) hence causes more conduction (Gervas et al., 2018). The replacement of Ni^2+^ ions with Co^2+^ ions in ferrite materials though increases the magnetic parameters such as coercivity (Patil et al., 2022). UV-Vis spectra reported by Hu et al. (2012) showed nearly a straight line which revealed that there is no absorption during the measurement hence no optical bandgap. So, the behavior of this material remains debatable. Being motivated by these analyses we also aimed to know the behavior.

Previously, NiCo_2_S_4_ nanostructures have been synthesized using a variety of well-known methods such as hydrothermal (Chen et al., 2013; Yu and Lin, 2016; Wei et al., 2017), solvothermal (Xin et al., 2020), electrodeposition (Chen et al., 2014; Cui et al., 2022), co-precipitation (Wang et al., 2016; Nan et al., 2018), etc. There is always a challenge to synthesize bimetallic compounds with desired morphology under relatively simpler conditions. Solventless thermolysis of elemental xanthates complexes are processed recently to prepare NiCo_2_S_4_ nanostructures (Khan et al., 2018; Shombe et al., 2021; Shombe et al., 2022). In this research work, we have successfully synthesized NiCo_2_S_4_ nanostructures using quite a simple, one-step, and inexpensive solvent-free (solid-state reaction route). We have studied the crystalline structure, built-in morphology, and a detailed understanding of its optical and electrical properties to determine the origin of this material and its extraordinary performance for various applications.

## Experimental details

For the synthesis of NiCo_2_S_4_, nickel acetate tetrahydrate (C_4_H_6_NiO_4_). 4H_2_O, cobalt acetate tetrahydrate (C_4_H_6_CoO_4_). 4H_2_O, and thiourea SC(NH_2_)_2_ were purchased from Sigma Aldrich and were of analytical grade, so used without any further purification. The stoichiometric amounts of these three precursors were mixed and ground in a pestle and mortar for 40 min to get a homogeneous mixture. In addition, we used a few drops of ethanol throughout the grinding process to improve the powder’s mixing. After that, the uniform mixture was put into the crucible for heat treatment. In a sequence, we have taken three random temperatures of 200°C, 300°C, and 400°C for the same reaction time (7 h). The possible chemical reactions during NiCo_2_S_4_ formation are suggested as follows.

Firstly, thiourea decomposes at a temperature of about 150°C (Ahmad et al., 2013a; Ahmad et al., 2013b; Ahmad et al., 2013c), as shown in the equation below.

$$SC{\left(NH_{2}\right)}_{2}\overset{\;∼150^{\circ }C\;}{\to }NH_{2}CN+H_{2}S.$$ (1)

Then *H*_*2*_ *S* reacts with nickel acetate tetrahydrate and cobalt acetate tetrahydrate forming NiCo_2_S_4_ with a few gases that are evaporated as byproducts during the reaction.

$$2\left[\left(C_{4\;}H_{6\;}CoO_{4}\right).4H_{2\;}O\right]+\left(C_{4\;}H_{6\;}NiO_{4}\right).4H_{2\;}O+{4H}_{2\;}S\to N_{i}{Co}_{2}S_{4}+25H_{2\;}+12{CO}_{2}.$$ (2)

**FIGURE 1:**
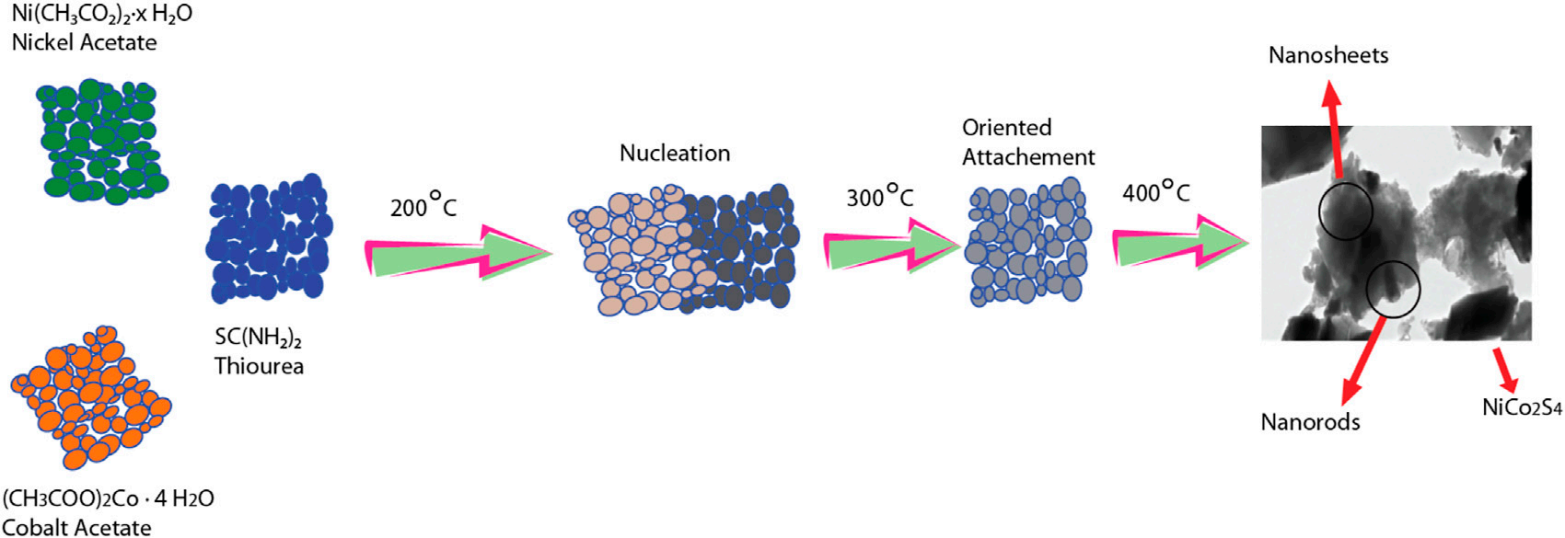
Schematic representation of steps involved e.g., nucleation, growth, and oriented attachment.

The steps involved in the material’s formation such as nucleation, growth, and oriented attachment are shown in Figure 1.

The powder sample prepared at 400°C was compacted into a 13 mm pellet using a hydraulic press machine at 1,200 Psi pressure for 10 min to study its electrical properties. The pellet was then sintered in an oven a 150°C for 2 h to make the material more compact. After that, we used the silver paste on both sides of the pellet to make an electrical contact.

The crystallographic structure of NiCo_2_S_4_ was studied by the x-rays diffraction (XRD) technique. The other structural parameter like crystallite size, lattice constants, texture coefficient, etc. were calculated from XRD data. The built-up surface morphology of the NiCo_2_S_4_ compound was analyzed using SEM and TEM respectively. The optical characteristics were investigated through UV-Vis. spectroscopy and DRS spectroscopy. The electrical properties of the prepared nanostructures at different temperatures were carried out through IV spectroscopy using the two-probe method.

## Results and discussions

**FIGURE 2:**
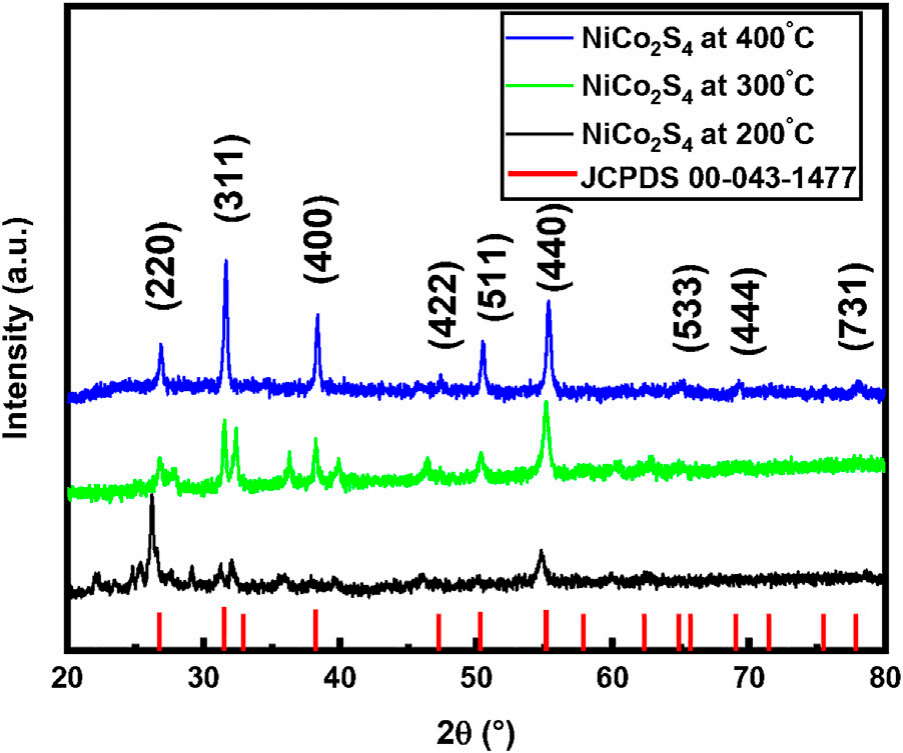
XRD spectrum of NiCo_2_S_4_ synthesized at different reaction temperatures.

Different attempts have been made to obtain their optimal crystalline nanostructures at varied reaction temperatures. Figure 2 shows the XRD spectrum of NiCo_2_S_4_ nanostructures prepared at 200°C, 300°C, and 400°C respectively. The XRD spectrum obtained at 200°C does not match fully with the standard pattern of NiCo_2_S_4_, indicating that its phase is incompletely formed. Only a few peaks were matched with JCPDS card number 00-043-1477. Many extra peaks were found in the spectrum which shows the possibility of the presence of some precursor elements due to incomplete reaction. To obtain the pure crystalline phase of NiCo_2_S_4_, we further treated the ground sample at 300 °C. However, we found some extra dominant peaks in the spectrum, so still, we believe that the pure phase of NiCo_2_S_4_ at 300°C was not obtained. When we increased the reaction temperature to 400°C we get the pure crystalline phase of NiCo_2_S_4_. The observed pattern is in good agreement with the standard JCPDS card number 00-043-1477 of NiCo_2_S_4_. No extra peak was noticed in the XRD pattern, which confirmed the purity and crystallinity of the sample. The XRD analysis confirms the cubic crystal structure of NiCo_2_S_4_. Using the lattice planes and d-spacing values the lattice constants were calculated. The average lattice constants are found to be a = b = c = 9.43 Å. Our calculated values are very much closer to the standard JCPDs card and with literature results that indicate the purity of our sample. The crystallite size is calculated through Scherrer’s relation. The average crystallite size of NiCo_2_S_4_ at T = 400°C is about 27 nm. We can also observe that the diffraction peaks got sharper and more dominant as the temperature was raised from 200°C to 400°C, indicating the improved crystallinity of the prepared material.

To check the preferable crystal growth direction, we calculated the texture coefficient of the prepared sample using the formula given below (Kumar et al., 2015).

$$T_{c}=\frac{\left(I_{hkl}/I_{r\left(hkl\right)}\right)}{\frac{1}{n}\Sigma_{n}\left(I_{hkl}/I_{r\left(hkl\right)}\right)}$$ (3)

where *I*_*hkl*_ is the intensity of the plane in the XRD spectrum of the sample, *I*_*r(hkl)*_ is the intensity of the corresponding plane in the reference pattern, and “*n*” is the number of peaks selected for the study. Moreover, an increase in texture coefficient from 1 is said to indicate a higher degree of preferred orientation along a given plane. The texture study shows that the NiCo_2_S_4_ nanostructures are highly textured along the (533) plane.

### Morphological analysis

**FIGURE 3:**
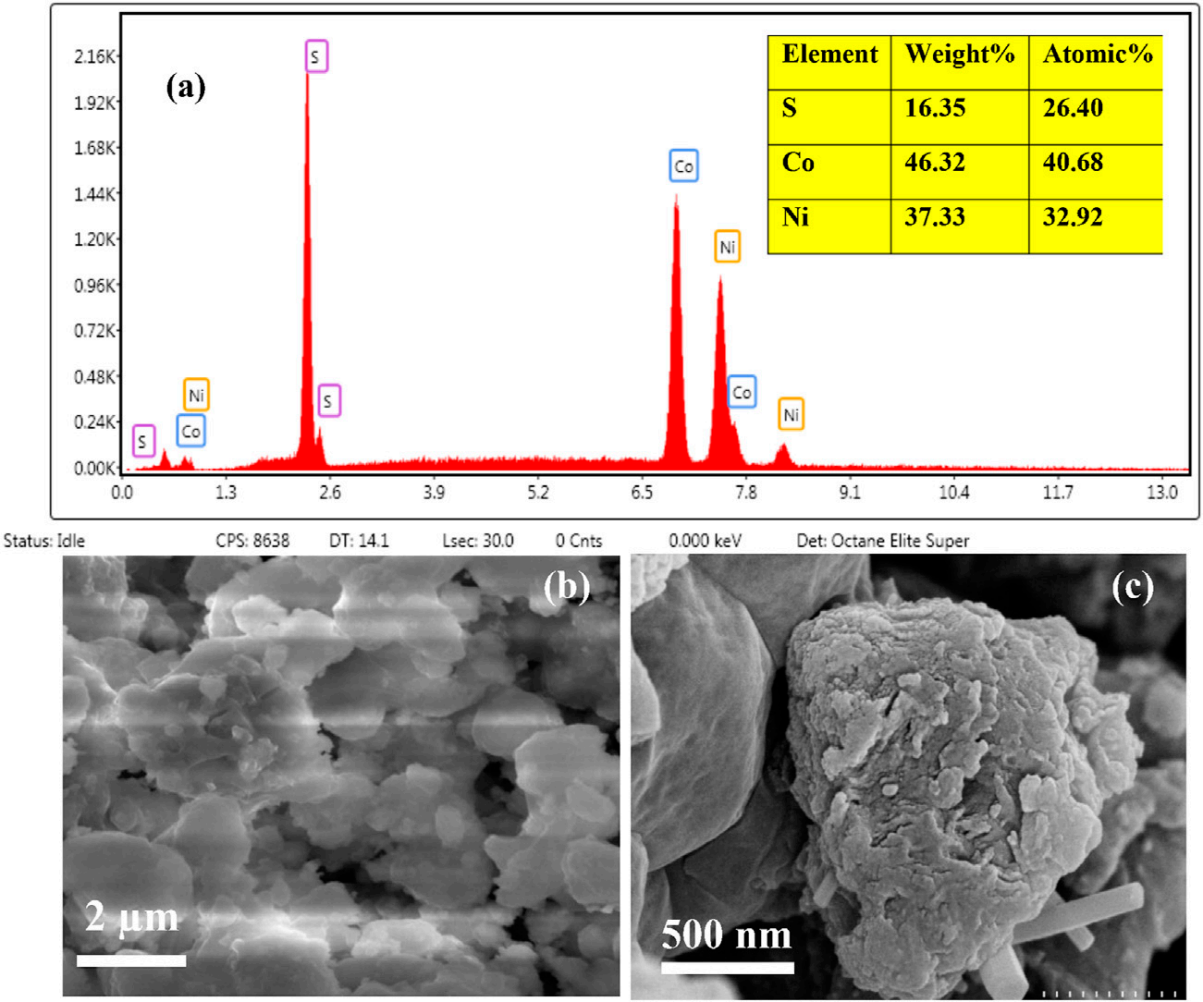
**(A)** EDX spectrum of NiCo_2_S_4_ **(B,C)** SEM images of NiCo_2_S_4_ at two different Resolutions.

Figure 3A shows the EDX spectrum of synthesized NiCo_2_S_4_ nanostructured. The presence of Co, Ni, and S elements and no other extra impurity peak in the graph confirms the purity of the prepared material. The detailed compositional analysis from a selected area is also shown. According to the EDX spectrum and table, the values are 26.4, 40.68, and 32.9 for S, Co, and Ni respectively. Therefore, the formula can be written as Ni_0.12_Co_0.46_S_0.12._ Figure 3B of the SEM image indicates that the agglomerates were assembled from randomly oriented bundles of nanoparticles adopting poly-disperse nanosheet-like morphology. Heating the acetate precursor resulted in the formation of agglomerated nanosheets of NiCo_2_S_4_. At a higher resolution in Figure 3C the agglomerates of nanostructures are more visible. Besides this agglomeration, we also see an interesting feature. Some nanorods of diameter ∼92 nm emerged as shown in the figure. This transition in the morphology may be caused due to high reaction temperature during sample preparation which breaks apart the agglomerates of nanoparticles and transforms them into nanorods.

**FIGURE 4:**
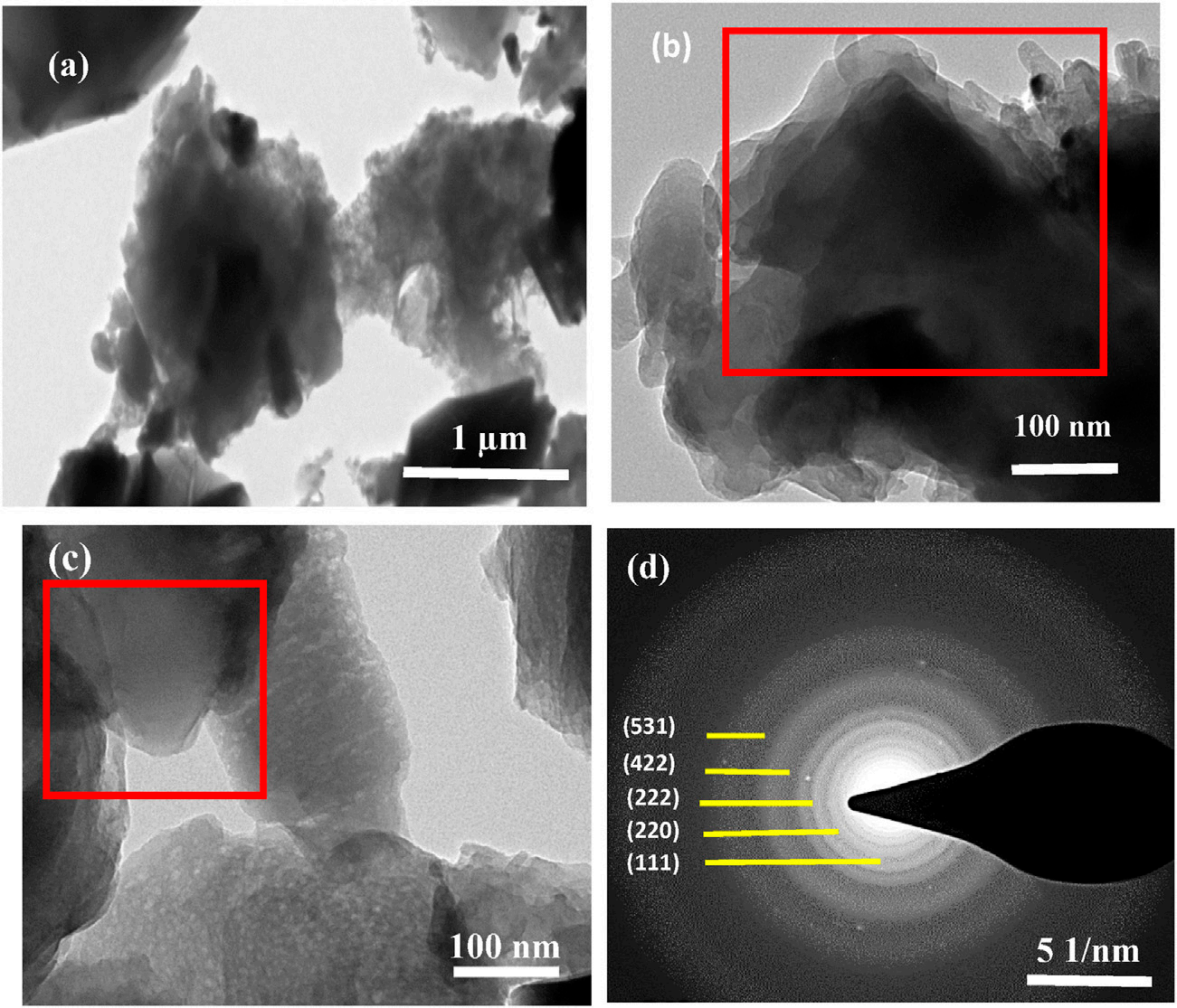
**(A–C)** TEM images of NiCo_2_S_4_ with different magnifications **(D)** SAED pattern of NiCo_2_S_4_.

To see the more in-depth morphology of the prepared sample, TEM (Figures 4A–C) was used. We can observe poly-sized sheet-like structures in TEM images more precisely shown in red boxes. Like in SEM analysis, we also spot some nanorods stripped into the agglomerates of nanosheets as shown in Figures 4A,B. We can see some grains/crystallites and grain boundaries in Figure 4C as well. Figure 4D shows the selected area electron diffraction (SAED) pattern of NiCo_2_S_4_. The diffraction rings associated with the polycrystalline nature of the prepared material are seen in the pattern. By using the Image-J software, we have successfully calculated the corresponding d–spacing of the rings are 5.68, 3.29, 2.69, 2.06, 1.55 Å corresponding to lattice planes (111), (220), (222). (422), and (531) respectively. These calculations were very close to those values calculated from XRD analysis and JCPDS card values. Again, from the SAED pattern, the lattice parameters are calculated as a = b = c = 9.47 Å. These calculated cell parameters are in good agreement with the literature results (Mane et al., 2021).

## Optical measurements

**FIGURE 5:**
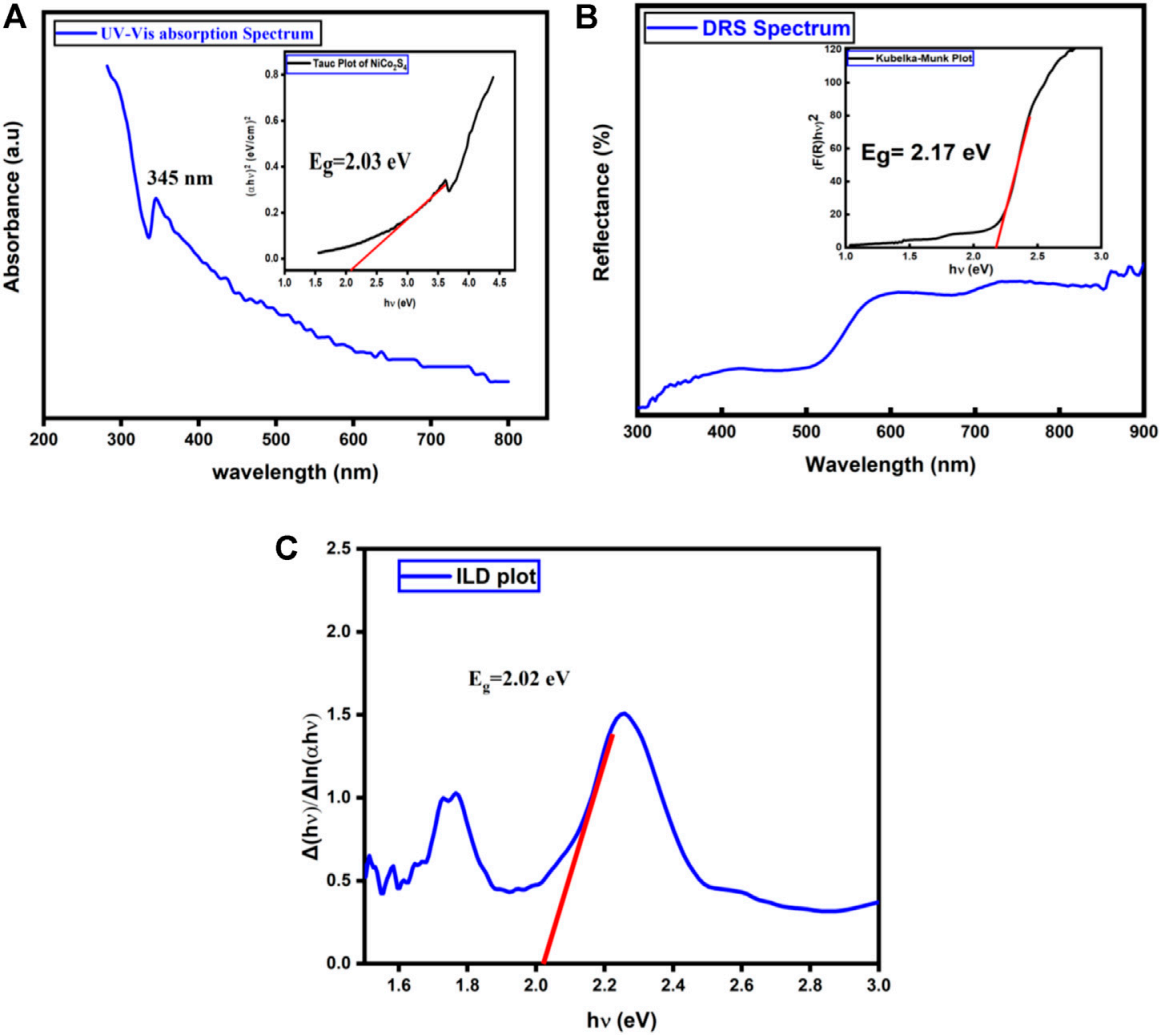
**(A)** Shows UV-Vis absorption spectrum and Tauc plot(inset) of NiCo_2_S_4_ nanostructures **(B)** DRS spectrum and Kubelka-Munk plot **(C)** Inverse logarithmic derivative of α*h* *v* as a function of hv (photon energy).

The optical analysis was studied using UV-Vis and DRS to investigate the bandgap of prepared NiCo_2_S_4_. Figure 5A shows the absorption spectrum with the inset of the Tauc plot. The absorption spectrum was recorded using the Shimadzu 1280 UV-Vis spectrophotometer. The absorption spectrum was recorded in the range of 200–800 nm at room temperature. We prepared a dilute suspension of a powder sample in deionized water to check the absorbance peak. The absorption peak arises near 345 nm. The band gap of the prepared nanomaterial was calculated using the Tauc equation (Granqvist, 1995; Gnanasekar et al., 2022; Raj et al., 2022; Rajeswari et al., 2022; Rokade et al., 2022).

$$\left(\alpha hv\right)=A\left(hv-E_{g}\right){\;}^{n}.$$ (4)

For the direct bandgap calculation of our specimen, a graph is plotted between ${\left(\alpha hv\right)}^{2}$ and photon energy ″ *h* $v^{\prime \prime }$ according to Beer-Lambert’s law (Wagh et al., 2022). The extrapolation of a linear region to the energy axis gives us a bandgap value. The calculated bandgap of NiCo_2_S_4_ is found to be 2.03 eV as shown in the figure. This band gap value is very much close to the already reported bandgap value of this material (Sarawutanukul et al., 2020).

Further, the optical bandgap of NiCo_2_S_4_ was also investigated through DRS. Figure 5B shows the variation of percentage reflectance against the wavelength of the incident source measured in the range of 300–900 nm. While the inset of Figure 5B shows the transformed Kubelka Munk plot obtained from the reflectance data. In DRS, the absorption coefficient ″α″ is replaced by Kubelka Munk or the re-emission function which is proportional to the absorption coefficient (*K*) and scattering coefficient (*S*).

$$\frac{k}{S}=\frac{\left(1-R_{\infty }\right)}{2R_{\infty }}\equiv F\left(R_{\infty }\right)$$ (5)

Where “*R*” is the measured reflected light. NiCo_2_S_4_ bandgap can be calculated by the transformed form of Eq. 4 in which ″α″ is replaced by $\prime \prime F\left(R_{\infty }\right)\prime \prime$ Kubelka Munk function (Kumar et al., 2013; Sundararajan et al., 2022a; Sundararajan et al., 2022b; Sathish Kumar et al., 2022).

$${\left(F\left(R_{\infty }\right)hv\right)}^{n}=A\left(hv-E_{g}\right)$$ (6)

The “*E*_*g*_” value of NiCo_2_S_4_ calculated from DRS spectroscopy is 2.17 eV as shown in the inset of Figure 5B. Again this value is very close to the bandgap value obtained from UV-Vis spectroscopy and previous literature (Zhao et al., 2020).

We also investigated the DRS spectra in more detail through the unique inverse logarithmic derivative (ILD) method (Pawlak and Al-Ani, 2019; Khan et al., 2021). By taking the natural logarithm on both sides of the Tauc Eq. 4:

$$\ln\left(ahv\right)=n\ln\left(A\right)+n\ln\left(hv-E_{g}\right).$$ (7)

Here “*A*” is eliminated because it does not depend on the photon energy “*hν*” and is also less significant practically than the “*n*” and “*E*_*g*_” factors. By differentiating Eq. 7 as a function of photon energy:

$$\frac{dln\left(\alpha h\nu \right)}{d\left(h\nu \right)}=\frac{n}{h\nu -E_{g}}.$$ (8)

By inverting Eq. 8

$$\frac{d\left(hv\right)}{d\ln\left(\alpha h\nu \right)}=\frac{hv-E_{g}}{n}.$$ (9)

Finally, by converting Eq. 9 into a numerical derivative:

$$\frac{\Delta \left(hv\right)}{\Delta \ln\left(\alpha h\nu \right)}=\frac{h\nu -E_{g}}{n}.$$ (10)

Then the graph of ″Δ(hv)/Δln(αhν)″ as a function of incident photon energy is plotted which gives us the value of “*E*_*g*_” by extrapolating the linear region into the energy axis. The bandgap of NiCo_2_S_4_ calculated from the “ILD” method is found to be 2.02 eV. Figure 5C shows the energy band gap graph of NiCo_2_S_4_ using the ILD method. From three different methods, the band gap value of NiCo_2_S_4_ is quite like one another and confirms the bandgap results.

As from both optical spectroscopies, NiCo_2_S_4_ has a specific optical bandgap like other semiconducting materials, therefore more detailed investigation of its metallic nature was done through temperature-dependent IV characteristics.

## Electrical measurements

**FIGURE 6:**
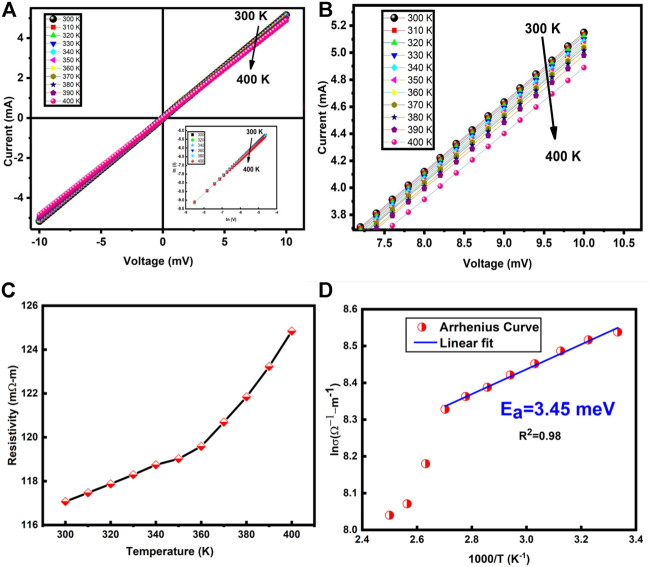
**(A)** IV response of NiCo_2_S_4_ at different temperatures and (inset) is the double logarithm graph of NiCo_2_S_4_ showing ohmic behavior. **(B)** Zoomed IV characteristics curves of NiCo_2_S_4_ **(C)** Resistivity varition with temperature **(D)** Arrhenius plot for the calculation of the activation energy.

The temperature-dependent electrical response of the prepared material is studied using the Keithley 2401 source meter. IV characteristics of NiCo_2_S_4_ nanostructures were measured in the temperature range 300–400 K using two probe method as shown in Figure 6A. When the temperature is increased from 300 to 400 K, the current decreases, showing metallic behavior. The *ln-ln* plot of IV characteristics is drawn and shown in the inset of Figure 6A. The slope of about ∼1 indicates the ohmic behavior. Figure 6B shows the zoomed part of IV characteristics. We can see clearly the decrease of conduction with the increase of temperature. The resistivity values are calculated from the IV data and are plotted against the temperature shown in Figure 6C. The increase in resistivity with temperature i.e., positive temperature coefficient of resistance indicates the metallic nature of NiCo_2_S_4_. This result of metallic behavior agreed very well with the recently reported literature (Xia et al., 2015). NiCo_2_S_4_ has a resistivity of the order of milli-ohm, making it a promising material for different energy storage and conversion applications.

Figure 6D shows the activation energy graph, plotted using the Arrhenius relation (Batool et al., 2020; Sankudevan et al., 2022) from well-fitted data in a low-temperature region. The activation energy is found to be 3.45 meV. This small value of activation energy is indicating the high cation activity (nickel and cobalt) of NiCo_2_S_4_. A comparative and detailed analysis of the literature work and our work is presented in Table 1.

**TABLE 1:** Comparison of the present study of NiCo_2_S_4_ with previously reported literature.

Synthesis (NiCo_2_S_4_)	Bandgap	Electrical behaviors	Morphology	References
Hydrothermal synthesis	—	Metallic behavior below the room temperature (5–300 K)	Urchin-like morphology	Xia et al. (2015)
A facile precursor transformation method	1.2 eV	Semiconducting with direct transition	Urchin-like nanostructure	Chen et al. (2013)
Solventless thermolysis synthesis	—	—	Agglomerated nanoparticles	Shombe et al. (2021)
Solvothermal process	1.71 eV with a direct bandgap transition	Prediction of metallic with no absorption in UV-Vis spectra	Quasi-spherical morphology	Du et al. (2014)
Electrodeposition method	—	—	Nanosheets Arrays	Chen et al. (2014)
Sulfurization of Ni and Co-based precursors	1.4 and 2.4 eV with a direct bandgap transition	—	3D urchin-like NiCo_2_S_4_	Sarawutanukul et al. (2020)
Solvothermal route	—	—	Mesoporous NiCo_2_S_4_ nanoparticles	Zhu et al. (2015)
Solvent-free solid-state route	2.02–2.17 eV with direct bandgap transition	Metallic nature above the room temperature (300–400 K)	Agglomerated sheet-like structures along with the growth of nanorods	Present work

## Conclusion

The crystalline cubic phase of NiCo_2_S_4_ is successfully achieved using a quite simple, one-step, and inexpensive solvent-free synthesis approach. The XRD analysis confirmed the successful formation of the cubic phase of NiCo_2_S_4_. Morphological analysis through SEM and TEM shows that agglomerated sheet-like structures with unusual growth of nanorods as well were built. The bandgap of NiCo_2_S_4_ is found to be 2.02–2.17 eV through the absorption and reflectance spectrum obtained from UV-Vis and DRS spectroscopy respectively. However, the electrical measurements at 300–400 K reveal that NiCo_2_S_4_ has a positive temperature coefficient of resistance which confirms the metallic nature of NiCo_2_S_4_. The superior electrical and optical properties, the small activation energy, low resistivity at room temperature, positive temperature coefficient of resistance, and better electronic conductivity make NiCo_2_S_4_ a potential option for numerous applications in diverse domains.

## Data Availability

The raw data supporting the conclusion of this article will be made available by the authors, without undue reservation.
